# Dental caries status in adults with sleep apnea – hypopnea syndrome

**DOI:** 10.4317/jced.59344

**Published:** 2022-03-01

**Authors:** Josep Pico-Orozco, Francisco-Javier Silvestre, Marina Carrasco-Llatas, Javier Silvestre-Rangil

**Affiliations:** 1 Department of Stomatology, Doctor Peset University Hospital, Valencia, Spain; 2 Ear, Nose and Throat (ENT) Department, Doctor Peset University Hospital, Valencia, Spain; 3 Special Care in Dentistry, Department of Stomatology, University of Valencia, Valencia, Spain

## Abstract

**Background:**

A study is made of dental caries in a group of adults with sleep apnea-hypopnea syndrome (SAHS), establishing comparisons with healthy individuals corresponding to the same population.

**Material and Methods:**

A case-control series was analyzed, including patients with recently diagnosed SAHS and individuals without SAHS. Dental examinations were made to record the DMF (decayed, missing, filled) dental score, and demographic, lifestyle and clinical data were collected.

**Results:**

A total of 114 participants (60 SAHS cases and 54 controls) were included in the study. Although the mean DMF score in the SAHS group was higher than in the control group (7.03 versus 4.81, respectively), the multivariate regression analysis did not find the difference to be statistically significant (*p*=0.351). However, a significant correlation was observed between the DMF score and age (r=0.41; *p*<0.001) and the apnea-hypopnea index (AHI)(r=0.31; *p*=0.003).

**Conclusions:**

Older age and greater severity of SAHS are associated to higher DMF scores. However, the diagnosis of SAHS alone does not influence dental caries status.

** Key words:**Dental caries, sleep apnea syndrome, oral health, DMF index.

## Introduction

Sleep apnea-hypopnea syndrome (SAHS) is increasingly common in our setting (1). Repeated total or partial interruption of breathing during sleep is associated to numerous comorbidities, particularly of a cardiovascular, metabolic and neurocognitive nature (2). More recent studies have also examined the relationship between SAHS and certain oral diseases such as periodontitis (3) and bruxism (4). Sleep apnea-hypopnea syndrome is more common in men, postmenopausal women and obese individuals (5). Likewise, certain anatomical and functional characteristics of the oral cavity may predispose to SAHS, such as macroglossia, an enlarged soft palate or uvula, or mouth breathing (6-8).

Dental caries is a chronic, multifactorial infectious disorder characterized by demineralization of the hard tissues of the tooth secondary to acid attack produced by the bacteria contained in the biofilm as a result of fermentation of the carbohydrates in the diet (9). Saliva plays a crucial role in protecting against caries (10). For this reason, patients with dry mouth are at an increased risk of developing dental caries (11).

The fact that patients with SAHS have an increased prevalence of dry mouth (12) could suggest that such individuals have a higher risk of suffering caries. Few studies have examined this possible relationship, however, and only one has been made in adults (13). Acar *et al*., in a Turkish study, concluded that SAHS does not affect dental condition (13). However, these results might not be extrapolable to our population, due to the socioeconomic and cultural differences between the two settings.

The present study based on the DMF (decayed, missing, filled) dental score was carried out to evaluate dental health in a group of adults recently diagnosed with SAHS in a public hospital in Valencia (Spain), establishing comparisons with healthy individuals corresponding to the same population.

## Material and Methods

-Study design and participants

A prospective, cross-sectional case-control study was carried out to compare dental caries in a group of patients with SAHS (group SAHS) versus a group of health individuals (control group). The participants in the study were recruited from the Ear, Nose and Throat, Pneumology and Dentistry Departments of Doctor Peset University Hospital (Valencia, Spain).

The SAHS group comprised patients with a recent (< 12 months) diagnosis of SAHS based on the polysomnography (PSG) or respiratory polygraphy (RP) findings, with an apnea-hypopnea index (AHI) > 15/h or AHI > 5/h plus associated symptoms (14). Based on the AHI, the patients were divided into three subgroups: mild SAHS (AHI 5-14/h), moderate SAHS (AHI 15-29/h) and severe SAHS (AHI ≥ 30/h). The control group in turn consisted of individuals without SAHS that were accompanying patients visiting the dental outpatient clinic.

Patients under 25 and over 75 years of age were excluded, as were those with fewer than 14 permanent teeth (excluding third molars), diabetic individuals with poor blood glucose control (glycosylated hemoglobin (HbA1c) ≥ 7%), subjects with acute infectious or inflammatory disorders, patients receiving treatment with antibiotics and/or systemic antiinflammatory drugs in the last 3 months, those subjected to dental treatment in the last 3 months, and pregnant or nursing women.

The study was carried out in abidance with the ethical principles of the Declaration of Helsinki regarding research in human subjects, and with the STROBE (Strengthening the Reporting of Observational Studies in Epidemiology) guidelines (15). Likewise, the study was approved by the Clinical Research Ethics Committee of our hospital (Ref.: 15/16). All the participants gave written informed consent to participation in the study.

-Data collection

An interview was first carried out to compile demographic data (age and gender) and a general medical history (past and current disease conditions, allergies, regular medication and toxic habits: smoking and alcohol). The subjects were also questioned about oral hygiene (frequency of brushing, date of last visit to the dentist, and presence of bleeding during brushing). The body mass index (BMI) was calculated for all participants as body weight (kg)/height (m)2.

With regard to the sleep history, the AHI was known in all the individuals belonging to the SAHS group, and was determined by PSG or RP (14). In the control group, SAHS was discarded based on clinical criteria: absence of snoring plus absence of daytime sleepiness as defined by a score of under 10 on the Epworth scale (16). Furthermore, home RP was carried out in one-half of these subjects, with the recording of AHI < 5/h in all cases.

All the dental explorations were made by a single experienced and calibrated dentist in the facilities of the Department of Dentistry (Doctor Peset University Hospital, Valencia, Spain), based on a standardized protocol and always under the same conditions. The evaluation of dental caries was based on the DMF (decayed, missing, filled) score (17) - the most widely used epidemiological index for establishing dental caries status in a given population group. The score sums the total number of decayed (D), missing (M) and filled teeth (F), and this in turn is divided by the number of subjects in the group. The presence of caries was taken to correspond to ICDAS codes 4, 5 and 6. A code of 4 defines a dark dentin shadow beneath the enamel layer; a code of 5 corresponds to an enamel cavity with visible dentin; and a code of 6 indicates an extensive cavity with visible dentin (18). In addition to the DMF score, we considered the value of each of the components (D, M and F) considered individually.

-Statistical analysis 

The descriptive statistical analysis reported the mean, standard deviation (SD), minimum and maximum, and median for continuous variables, while categorical variables were expressed as absolute frequencies and percentages. Analysis of the homogeneity of the groups was based on the Student t-test for independent samples (continuous variables) and the chi-squared test (categorical variables). Comparisons between groups were made using the Student t-test for independent samples. Analysis of covariance (ANCOVA) was performed to evaluate the mean caries scores according to the group involved, adjusting for variables of the subject profile. Correlations between different parameters and the DMF score were established using the Pearson correlation coefficient.

Statistical significance was considered for *p* < 0.05. The SPSS version 18.0 statistical package (IBM SPSS Statistics Inc., Chicago, IL, USA) was used throughout.

## Results

A total of 114 subjects were studied (57 men and 57 women), with a mean age of 52.9 ± 10.2 years (range 27-71). The participants were divided into two groups according to whether SAHS was diagnosed or not (n=60 and n=54, respectively). The discrepancy in size between the two groups (both of which initially comprised 57 individuals) was due to the fact that at the time of control RP, three of the controls were found to have SAHS and therefore changed group. In the SAHS group, the mean AHI was 24.9/h. Specifically, 15 subjects (25%) had mild SAHS (AHI: 5-14/h), 24 (40%) moderate SAHS (AHI: 15-29/h), and 21 (35%) severe SAHS (AHI ≥ 30).

Table 1 shows some of the demographic and clinical characteristics of the study sample. The SAHS patients were somewhat older (SAHS: 55.4 ± 8.2 years; control: 50.1 ± 11.5 years; *p*=0.007), and had a higher BMI (SAHS: 29.9 ± 4.1 kg/m2; control: 23.9 ± 2.9 kg/m2; *p*<0.001). In contrast, the two groups were homogeneous in terms of gender distribution (*p*=0.708), diabetes mellitus (*p*=0.056), smoking (*p*=0.256) and alcohol intake (*p*=0.248). With regard to oral hygiene, the patients with SAHS showed a lower brushing frequency (*p*=0.023), and had visited the dentist more often in the last year (*p*=0.014). In relation to the presence of bleeding during brushing, the Figures were seen to be similar in both groups (SAHS: 41.7%; control: 38.9%; *p*=0.763).

**Table 1:**
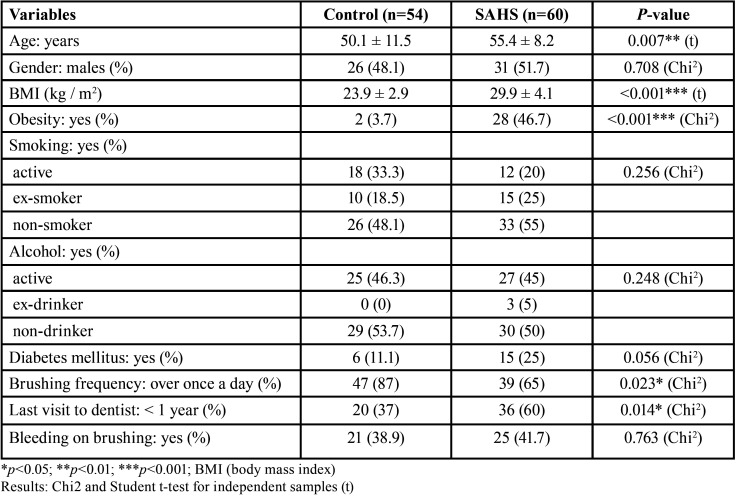
General characteristics of the study sample (SAHS versus control).

**Figure 1:**
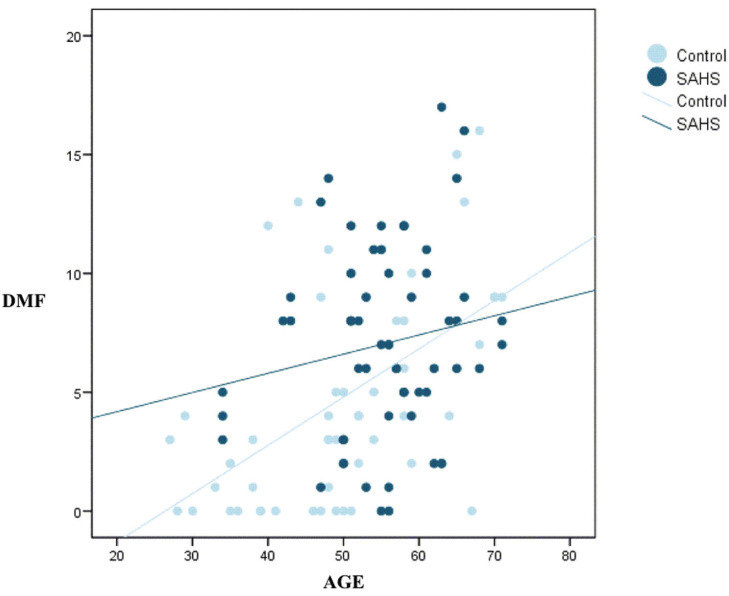
Relationship between DMF score and age (SAHS versus control).

The mean DMF score in the SAHS group was greater than in the control group (7.03 versus 4.81, respectively). This comparative increase was attribuTable to a larger number of teeth with caries (D) and missing teeth (M) due to caries (Table 2). The Student t-test for independent samples indicated a higher mean DMF score in the SAHS group (*p*=0.007). However, the multivariate regression model showed the true determinant of the DMF score to be the age of the individual (*p*<0.001), thereby neutralizing the purported influence of the diagnosis of SAHS (*p*=0.351) (Fig. 1). In other words, older age was associated to higher DMF scores. For one same age, no differences in DMF score were observed between the subjects with and without SAHS. With regard to the individual components of the index, no significant association was observed between the mean number of caried teeth (D) and age (*p*=0.115).

**Table 2:**
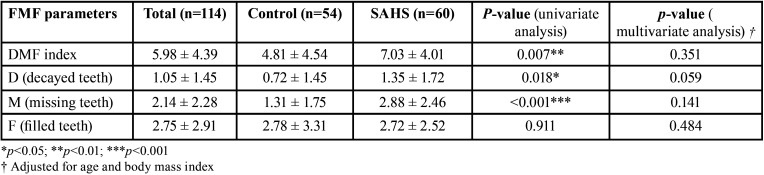
DMF scores (SAHS versus control).

**Figure 2:**
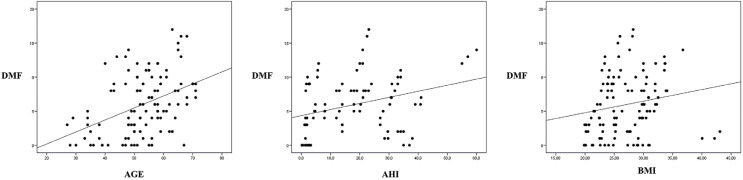
Bivariate correlations between DMF.

Table 3 describes the correlations between the DMF index (and its individual components) and AHI, age and BMI. The DMF score showed a statistically significant correlation to AHI (r=0.31; *p*=0.003) and age (r=0.41; *p*<0.001), and a close to significant correlation to BMI (r=0.18; *p*=0.054) (Fig. 2).

**Table 3:**
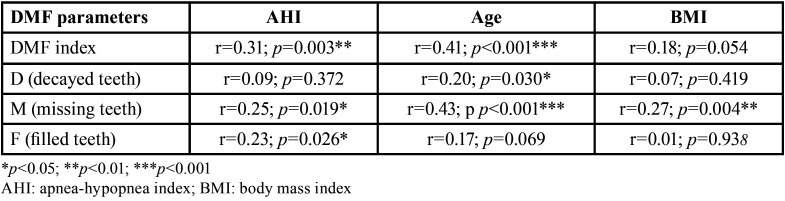
Correlation between DMF score and AHI score, age and BMI.

## Discussion

In the present study, worse caries disease was observed in the population with SAHS than in the healthy subjects, though this difference is more attributable to the fact that the individuals with SAHS were comparatively older, than to the diagnosis of SAHS itself. In addition to age, a direct correlation was observed between the DMF score and AHI. Accordingly, the severity of dental caries was seen to increase with older age and greater severity of SAHS. To the best of our knowledge, this is the first study to describe a correlation between the DMF score and the apnea-hypopnea index (AHI).

Oral health in patients with SAHS has been previously studies in positive airway pressure patients (continuous positive airway pressure (CPAP), bilevel positive airway pressure (BiPAP)) (19). In the latter study, no differences were recorded with respect to the controls. However, the authors did not evaluate any dental caries index. Mouth breathing, which is very common in patients with SAHS, results in increased dry mouth (20), and the latter is known to have a negative impact upon dental health (10). The role of saliva in protecting against caries can be explained in terms of the dilution and elimination (clearance) of sugars and other substances; buffer action; dental hard tissue demineralization – remineralization balancing effects; and antimicrobial activity (21). To date, only three studies have explored the possible association between caries and SAHS: two in the pediatric population (22,23) and one in adults (13).

In relation to the subjects investigated in our study, i.e., adults with SAHS, Acar *et al*. (13) evaluated the dental condition of 291 patients diagnosed on the basis of the polysomnographic findings with either SAHS (250 patients) or primary snoring disorder (40 patients) – the latter constituting the control group. Both groups yielded a very similar DMF score (mean 10.6 ± 6.50). The authors possibly would have obtained different results if the controls had been non-snoring healthy individuals, as in our study, since snorers are less likely to be mouth breathers. In support of this, the mentioned investigators recorded a positive correlation between the DMF score and the duration of snoring. In contrast to our own study, they recorded no correlation between the DMF score and AHI - though both studies coincided in identifying a direct correlation between the DMF score and the age of the subject. These results referred to age underscore what we regard as one of the inconveniences of the DMF index, namely the fact that it not only measures current caries status but also past caries experience; accordingly, since older individuals are more likely to have experienced caries at some point in the past, they consequently also yield a higher DMF score. This aspect is important in patients with SAHS, since the risk of the syndrome is known to increase with age (5). Another inconvenience of the DMF index is referred to component M (missing teeth), since patients are not always able to recall whether a given tooth was lost because of caries or as a consequence of some other circumstance (periodontitis, traumatism, orthodontics). In this respect, the higher prevalence of periodontitis (and the consequent greater number of missing teeth) in patients with SAHS (3) could mask the impact of this variable. The same problem can occur with regard to component F (filled teeth), since caries disease is not necessarily the reason for dental restoration in all cases. Accordingly, the increased prevalence of bruxism and dental wear in these patients (4,24) could justify the greater need for restorative treatments, with a consequent greater number of fillings.

On considering the individual components of the DMF score, component D (decayed teeth) was not significantly influenced by age (*p*=0.115), though a certain trend towards significance was observed in the case of a diagnosis of SAHS (*p*=0.059). In this respect, the lesser brushing frequency recorded in the SAHS population of our series (*p*=0.023) might lie behind this observed trend. Nevertheless, we observed no correlation between component D and the AHI (*p*=0.372); the correlation between the DMF score and the AHI was thus established at the expense of the number of teeth missing because of caries (*p*=0.019) and of the number of filled teeth (*p*=0.026).

In conclusion, caries status in the patients diagnosed with SAHS was poorer than in the healthy controls, but this difference was more conditioned by the fact that the patients with SAHS were comparatively older than by the actual diagnosis of the syndrome. Likewise, in the patients with SAHS, a statistically significant association was observed between the DMF score and age and the AHI score.

## References

[1] Heinzer R, Vat S, Marques-Vidal P, Marti-Soler H, Andries D, Tobback N (2015). Prevalence of sleep-disordered breathing in the general population: the HypnoLaus study. Lancet Respir Med.

[2] Dempsey JA, Veasey SC, Morgan BJ, O'Donnell CP (2010). Pathophysiology of sleep apnea. Physiol Rev.

[3] Gunaratnam K, Taylor B, Curtis B, Cistulli P (2009). Obstructive sleep apnoea and periodontitis: a novel association?. Sleep Breath.

[4] Hosoya H, Kitaura H, Hashimoto T, Ito M, Kinbara M, Deguchi T (2014). Relationship between sleep bruxism and sleep respiratory events in patients with obstructive sleep apnea syndrome. Sleep Breath.

[5] Young T, Skatrud J, Peppard PE (2004). Risk factors for obstructive sleep apnea in adults. JAMA.

[6] Ruangsri S, Jorns TP, Puasiri S, Luecha T, Chaithap C, Sawanyawisuth K (2016). Which oropharyngeal factors are significant risk factors for obstructive sleep apnea? An age-matched study and dentist perspectives. Nat Sci Sleep.

[7] Petrou-Amerikanou C, Belazi MA, Daskalopoulou E, Vlachoyiannis E, Daniilidou NV, Papanayiotou PC (2005). Oral findings in patients with obstructive sleep apnea syndrome. Quintessence Int.

[8] Kale SS, Kakodkar P, Shetiya SH (2018). Assessment of oral findings of dental patients who screen high and no risk for obstructive sleep apnea (OSA) reporting to a dental college - A cross sectional study. Sleep Sci.

[9] Selwitz RH, Ismail AI, Pitts NB (2007). Dental caries. Lancet.

[10] Brosky ME (2007). The role of saliva in oral health: strategies for prevention and management of xerostomia. J Support Oncol.

[11] Su N, Marek CL, Ching V, Grushka M (2011). Caries prevention for patients with dry mouth. J Can Dent Assoc.

[12] Oksenberg A, Froom P, Melamed S (2006). Dry mouth upon awakening in obstructive sleep apnea. J Sleep Res.

[13] Acar M, Türkcan İ, Özdaş T, Bal C, Cingi C (2015). Obstructive sleep apnoea syndrome does not negatively affect oral and dental health. J Laryngol Otol.

[14] Sateia MJ (2014). International classification of sleep disorders-third edition: highlights and modifications. Chest.

[15] von Elm E, Altman DG, Egger M, Pocock SJ, Gotzsch PC, Vandenbroucke JP (2008). The strengthening the reporting of observational studies in epidemiology (STROBE) statement: guidelines for reporting observational studies. J Clin Epidemiol.

[16] Johns M, Hocking B (1997). Daytime sleepiness and sleep habits of Australian workers. Sleep.

[17] Klein H, Palmer CE, Knutson JW (1938). Studies on dental caries. Dental status and dental needs of elementary school children. U.S. Public-Health Rep.

[18] Gugnani N, Pandit IK, Srivastava N, Gupta M, Sharma M (2011). International Caries Detection and Assessment System (ICDAS): A New Concept. Int J Clin Pediatr Dent.

[19] Carra MC, Thomas F, Schmitt A, Pannier B, Danchin N, Bouchard P (2016). Oral health in patients treated by positive airway pressure for obstructive sleep apnea: a population-based case-control study. Sleep Breath.

[20] Ruhle KH, Nilius G (2008). Mouth Breathing in Obstructive Sleep Apnea Prior to and During Nasal Continuous Positive Airway Pressure. Respiration.

[21] Llena-Puy C (2006). The Rôle of Saliva in Maintaining Oral Health and as an Aid to Diagnosis. Med Oral Patol Oral Cir Bucal.

[22] Al-Hammad NS, Hakeem LA, Salama FS (2015). Oral health status of children with obstructive sleep apnea and snoring. Pediatr Dent.

[23] Tamasas B, Nelson T, Chen M (2019). Health and oral health-related quality of life in children with obstructive sleep apnea. J Clin Sleep Med.

[24] Durán-Cantolla J, Alkhraisat MH, Martínez-Null C, Aguirre JJ, Guinea ER, Anitua E (2015). Frequency of obstructive sleep apnea syndrome in dental patients with tooth wear. J Clin Sleep Med.

